# Potassium Iodide and Citrates in Pneumonia

**Published:** 1909-04-03

**Authors:** 


### POTASSIUM IODIDE AND CITRATES IN PNEUMONIA.

The pneumococcus is an organism which exhibits remarkable variability in its virulence in different seasons; hardly a death from pneumonia occurs in some years, whilst fatalities are distressingly numerous in others. Whatever drug happens to have been widely used in the treatment of pneumonia cases in a favourable year is apt to receive too much credit for the cures; when it is tried in similar cases in the bad years it proves to be no better than many of the other remedies that have been tried. It is next to impossible, therefore, to say precisely what value is to be attached to any particular line of drug treatment in pneumonia. Probably if nursing is efficient, if pain is relieved, thirst assuaged, and sleep encouraged and ensured, nearly, if not quite, as large a percentage of recoveries will be obtained when no special remedies are given as when this or that particular drug is used in the belief that it does specific good in these cases. Nevertheless there are many who advocate one particular line of treatment or another with firm belief; amongst such methods is that by iodide of potassium and the citrates of potassium and ammonium, originated, we believe, by Dr. Ewart. An initial dose of calomel having been given, the following mixture is employed:—

| | | |
|---|---|---|
| ℞ | Potassii iodidi ... ... ... ... | gr. iv. |
| | Potassii citratis ... ... ... ... | gr. x. |
| | Liquoris ammonii citratis ... ... | ʒss. |
| | Excip. ad ... ... ... ... | ℥j. |

Some authorities lay stress on giving this very frequently to start with, say every hour for the first six hours, then every two hours till next day, and thereafter four hourly until after the crisis. Others give the mixture four hourly from the commencement. Others, again, believe in giving a still larger dose of the ammonium citrate, even when administering it hourly. If one is not already wedded to a favourite prescription, the above seems well worthy of trial.

